# Effectiveness of an e-Bug–based antimicrobial resistance education intervention: A prospective quasi-experimental study among Grade 12 students in Mogadishu, Somalia

**DOI:** 10.1016/j.ijregi.2025.100771

**Published:** 2025-09-25

**Authors:** Shafie Abdulkadir Hassan, Maryan Jamal Isak, Abdullahi Mohamed Osman, Yonis Mohamud Hassan, Ahmed Mohamed Ahmed, Sara Ali Mire, Kassim Abdi Jimale, Abdifetah Ibrahim Omar, Nur Rashid Ahmed

**Affiliations:** 1 Faculty of Medicine and Health Science, Jamhuriya University of Science and Technology (JUST), Mogadishu, Somalia; 2 Center for Antimicrobial Resistance Research, Jamhuriya University of Science and Technology, Mogadishu, Somalia

**Keywords:** e-Bug, Antimicrobial resistance, AMR, Health education, Somalia, Secondary students

## Abstract

**Objectives:**

Antimicrobial resistance (AMR) is a severe global health threat, with a disproportionate impact on low-income countries like Somalia. This study aimed to evaluate the effectiveness of the e-Bug educational module in improving knowledge and understanding of AMR among secondary school students in Mogadishu, Somalia, a setting with limited public awareness and a high burden of infectious diseases.

**Methods:**

A prospective quasi-experimental pre-post study was conducted among 384 Form four (Grade 12) students in Mogadishu. A structured questionnaire assessed knowledge of microbiology, hygiene, and AMR before and after the implementation of the e-Bug educational module. The intervention consisted of standardized, interactive lessons on AMR, hygiene, and infection prevention. Changes in knowledge were analyzed using descriptive statistics, and the McNemar-Bowker test was used to determine the statistical significance of the change in responses from pre-test to post-test.

**Results:**

Participants were predominantly male (54.2%), with most aged 16-18 years (51.6%). Baseline data revealed strong knowledge of general hygiene, such as handwashing (93.5%), but notable misunderstandings regarding AMR. Notably, 61.5% of students incorrectly believed antibiotics are effective against viral infections like influenza. Following the e-Bug intervention, a statistically significant improvement in overall AMR knowledge was observed (*P* = 0.003). Post-test results showed enhanced understanding of key AMR concepts, including a 9.6% increase (from 84.4% to 94.0%) in the awareness that antibiotic misuse drives resistance. However, the misconception that antibiotics treat viral infections remained prevalent (58.3%).

**Conclusions:**

The e-Bug educational module is an effective tool for improving AMR knowledge among secondary school students in Mogadishu. However, the persistence of critical misconceptions, particularly regarding the use of antibiotics for viral illnesses, highlights the need for sustained and reinforced educational efforts. Integrating the e-Bug module into the national curriculum and developing targeted public health campaigns are recommended to address these deep-seated misunderstandings and combat the growing threat of AMR in Somalia.

## Highlights

• The e-Bug module significantly boosted overall antimicrobial resistance knowledge (*P* = 0.003).
• Awareness of antibiotic misuse causing resistance increased from 84.4% to 94.0%.
• A major misconception remains: 58.3% think antibiotics treat viral infections.
• Baseline: Strong hygiene knowledge, but 61.5% initially held false antimicrobial resistance beliefs.

## Introduction

Antimicrobial resistance (AMR) is a leading public health threat of the 21st century, responsible for over 1.27 million deaths globally in 2019 and associated with nearly 5 million deaths [1]. The burden is highest in low- and middle-income countries, with projections estimating 10 million annual deaths from AMR by 2050 if urgent action is not taken [2].

In Somalia, the AMR situation is particularly critical [3]. Decades of instability have resulted in a fragmented health system [4]. This system is characterized by unregulated access to antibiotics, a lack of diagnostic capacity, and minimal public awareness [5]. This environment fosters the inappropriate use of antibiotics for self-limiting or viral conditions, driving the emergence and spread of resistant pathogens [6]. In 2019, an estimated 8400 deaths in Somalia were directly attributable to AMR, with over 32,700 deaths linked to AMR-related complications [7].The widespread availability of antibiotics without a prescription and the prevalence of substandard drugs further exacerbates this crisis [8].

Educating the next generation of antibiotic users is a cornerstone of the global strategy to combat AMR [9]. School-based interventions can shape lifelong health behaviors and empower young people to become “change agents” within their families and communities [10]. The e-Bug program was developed by the United Kingdom (UK) Health Security Agency and is a free, evidence-based educational resource [11]. It is designed to teach children and adolescents about microbes, hygiene, and the responsible use of antibiotics [12]. Its effectiveness has been demonstrated in various high- and middle-income settings, showing significant improvements in student knowledge [13].

Knowledge of AMR among young people in Somalia remains critically underexplored. To date, no published study has assessed baseline knowledge in this population or evaluated the feasibility of implementing a proven educational program, such as e-Bug, within the country’s fragile context. This study aims to fill that critical gap by evaluating the effectiveness of the e-Bug educational module in improving knowledge of AMR among secondary school students in Mogadishu, Somalia.

## Materials and methods

### Study design and setting

A prospective quasi-experimental pre-post study was conducted on Grade 12 students from selected schools in Mogadishu, the capital of Somalia from February to July 2025.

### The structure of the Somali education system

Somalia’s education system is structured on an 8-4 model of primary and secondary schooling, supplemented by alternative basic education programs, and is defined by a decentralized governance structure with a dominant private sector. This dynamic is most evident in the capital, Mogadishu, which hosts the nation’s highest concentration of schools, with 96% being privately managed and all situated in urban areas. A significant 85% of schools in the capital are integrated, offering both primary and secondary levels.

### Selection of study participants and criteria

**Figure 1:**
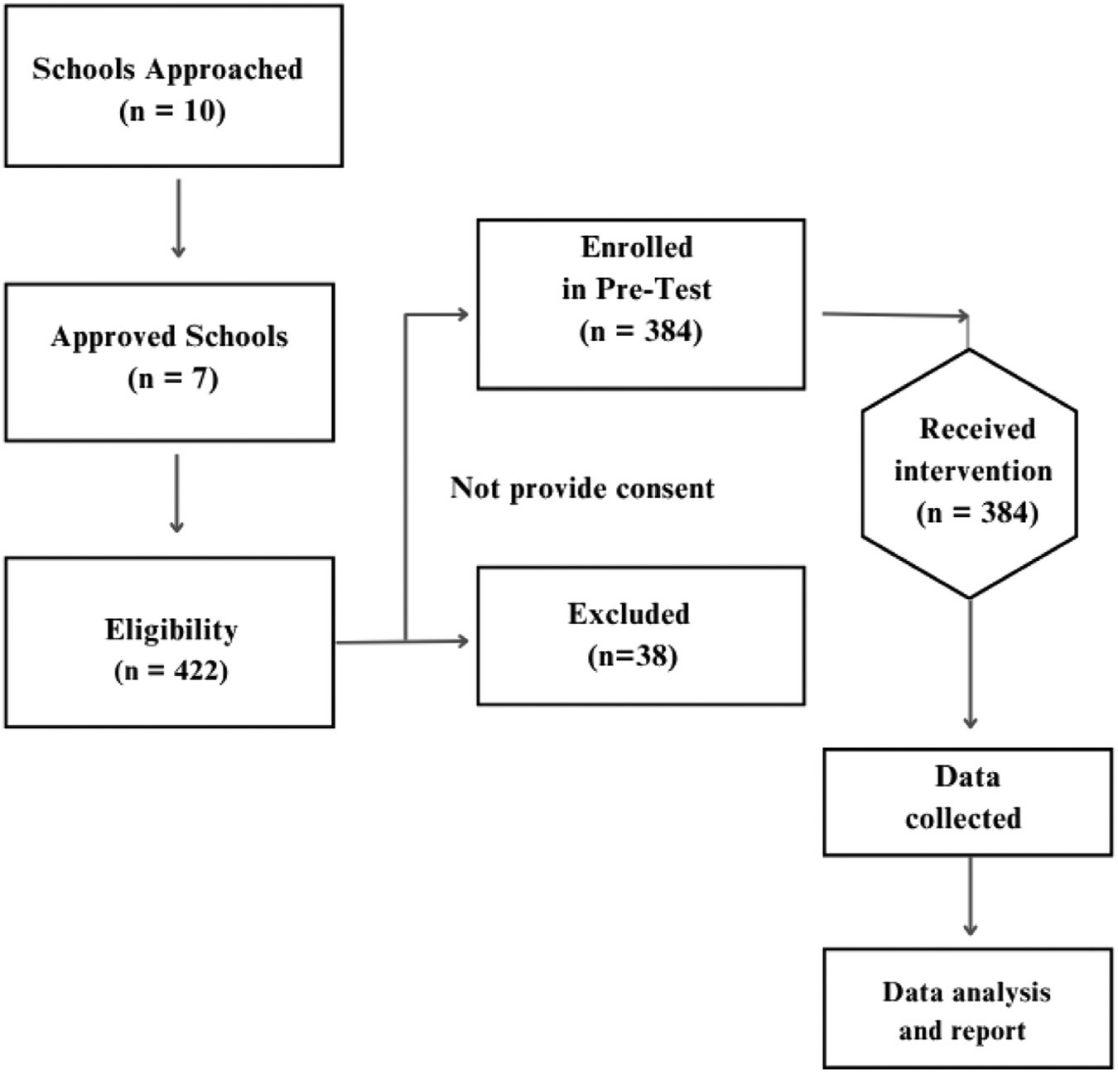
Flow of participants through the study.

The study was conducted in Mogadishu, where 10 schools, including both public and private institutions, were initially approached to participate. Following administrative engagement, seven of these schools granted official permission to proceed. Before commencing, ethical clearance was secured from the Jamhuriya Research Ethics Committee, and formal approval was obtained from the Banadir Regional Education Directorate. During an initial visit to the seven confirmed schools, study information sheets and a total of 500 parental consent forms were distributed to students, and a focal teacher was assigned at each site to facilitate coordination. The primary inclusion criterion was active enrollment in one of the participating schools. Of the forms distributed, 422 were returned with signed parental consent. On a second visit, student assent was obtained from these 422 participants, who were then included in the pre-test, forming the initial study cohort. Following the health education intervention, a post-test was administered. At this stage, 38 students were excluded from the final analysis due to attrition, as they were absent or had missed the intervention. Consequently, the final sample size included in the data analysis comprised 384 students (Figure 1).

### Intervention

**Figure 2:**
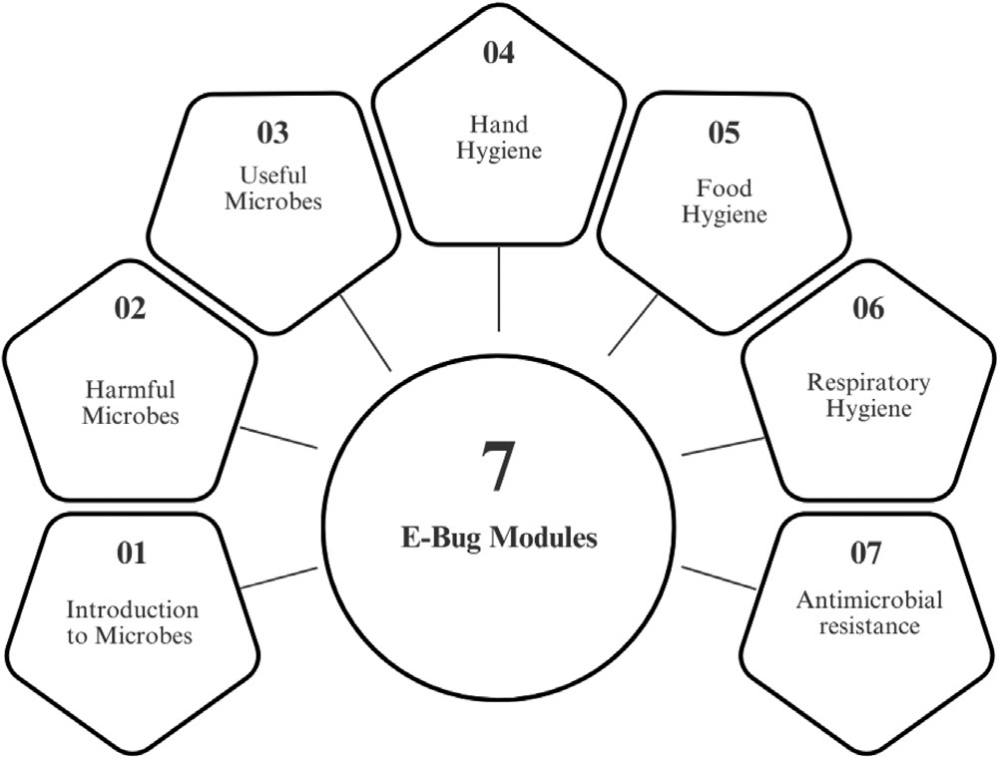
Topics addressed during the intervention using e-Bug modules.

The intervention consisted of the e-Bug educational module designed for Key Stage 4 students (14-16 years). Facilitators completed the Continuing Professional Development (CPD) certified e-Bug Health Educator Training course online via FutureLearn over a 3-week period, developed by the British Society for Antimicrobial Chemotherapy and the UK Health Security Agency [14]. The content focused on fundamental microbiology, routes of infection transmission, personal hygiene (e.g., handwashing, cough etiquette), the difference between bacteria and viruses, and the correct use of antibiotics and the development of AMR. The module used interactive activities and group discussions to engage students (Figure 2).

### Data collection

A structured, self-administered questionnaire was used for data collection. The questionnaire was administered twice: once before the intervention (pre-test) to establish baseline knowledge and again immediately after the 2-week intervention (post-test) to measure any change. The instrument collected sociodemographic data (age, sex) and assessed knowledge with 10 true/false questions covering key topics: visibility of microbes, role of handwashing, antibiotic effectiveness against bacteria vs viruses, and the link between antibiotic misuse and AMR.

### Statistical analysis

Data were entered and analyzed using SPSS (Version 24). Descriptive statistics (frequencies and percentages) were used to summarize sociodemographic characteristics and the proportion of correct/incorrect answers for each knowledge question in both the pre-test and post-test. To evaluate the effectiveness of the intervention, the McNemar-Bowker test, a non-parametric test for paired categorical data, was used to assess the statistical significance of the change in the distribution of responses from pre-test to post-test. A *P*-value of <0.05 was considered statistically significant.

## Results

### Sociodemographic characteristics

A total of 384 Form four (Grade 12) students participated in the study. As shown in Table 1, most participants were male (54.2%) and were in the 16 to 18-year age group (51.6%).

**Table 1:** Socio-demographic characteristics of study participants (n = 384).

Variable	Category	Frequency	Percentage
Age/years	16-18	198	51.6
	19-21	172	44.8
	22-24	14	3.6
Sex	Male	208	54.2
	Female	176	45.8

### Baseline (pre-test) knowledge

Pre-intervention results indicated a strong baseline understanding of general hygiene principles. Over 90% of students correctly identified the importance of covering coughs and sneezes (91.4%) and frequent handwashing (93.5%). However, significant misconceptions were prevalent (Table 2). A majority incorrectly believed that viruses are the largest type of microbes (74.2%) and that all microbes are harmful (77.3%). Critically, 61.5% of students believed that antibiotics are effective for treating viral infections like influenza.

**Table 2:** Pre-test knowledge on microbes, hygiene, and antimicrobial resistance among secondary school students.

No.	Statement	True *N* (%)	False *N* (%)
Q1	Microbes are visible to the naked eye.	76 (19.8)	308 (80.6)
Q2	All microbes on hands are beneficial.	87 (22.7)	297 (77.3)
Q3	Viruses are the largest type of microbe.	285 (74.2)	99 (25.8)
Q4	Antibiotics treat bacterial infections (e.g., tuberculosis).	268 (69.8)	116 (30.2)
Q5	Antimicrobial resistance occurs when microbes resist medicines.	295 (76.8)	89 (23.2)
Q6	Always cover coughs and sneezes.	351 (91.4)	33 (8.6)
Q7	Frequent handwashing prevents microbe spread.	359 (93.5)	25 (6.5)
Q8	Microbes are used to make bread and yogurt.	261 (68.0)	123 (32.0)
Q9	Antibiotics are effective against viruses (e.g., influenza).	236 (61.5)	148 (38.5)
Q10	Overuse/misuse of antibiotics leads to antimicrobial resistance.	324 (84.4)	59 (15.4)

### Post-intervention (post-test) knowledge

Following the e-Bug intervention, knowledge improved across most areas (Table 3). Awareness of the importance of covering coughs (97.7%) and handwashing (97.4%) was further reinforced. Understanding that microbes are not visible to the naked eye increased from 80.6% to 93.8%. Most importantly, the percentage of students correctly identifying that overuse and misuse of antibiotics lead to AMR increased from 84.4% to 94.0%. Despite this, the misconception that antibiotics are effective against viral infections remained, with 58.3% still holding this incorrect belief.

**Table 3:** Post-test knowledge on microbes, hygiene, and antimicrobial resistance among secondary school students.

No.	Statement	True *N* (%)	False *N* (%)
Q1	Microbes are visible to the naked eye.	24 (6.3)	360 (93.8)
Q2	All microbes on hands are beneficial.	64 (16.7)	320 (83.3)
Q3	Viruses are the largest type of microbe.	249 (64.8)	135 (35.2)
Q4	Antibiotics treat bacterial infections (e.g., tuberculosis).	292 (76.0)	92 (24.0)
Q5	Antimicrobial resistance occurs when microbes resist medicines.	327 (85.2)	57 (14.8)
Q6	Always cover coughs and sneezes.	375 (97.7)	9 (2.3)
Q7	Frequent handwashing prevents microbe spread.	374 (97.4)	10 (2.6)
Q8	Microbes are used to make bread and yogurt.	345 (89.8)	39 (10.2)
Q9	Antibiotics are effective against viruses (e.g., influenza).	224 (58.3)	160 (41.7)
Q10	Overuse/misuse of antibiotics leads to antimicrobial resistance.	361 (94.0)	23 (6.0)

### Effectiveness of the intervention

The McNemar-Bowker test showed a statistically significant difference in the overall pattern of responses between the pre-test and post-test (X² [3] = 13.62, *P* = 0.003). This indicates that the e-Bug educational program led to a significant improvement in students’ knowledge of AMR. Crosstabulation of knowledge levels (categorized as Poor, Moderate, Good) showed a clear shift, with a notable number of students moving from moderate to good knowledge post-intervention (Table 4).

**Table 4:** Crosstabulation of knowledge levels pre- and post-intervention with McNemar-Bowker test results.

Pre-test knowledge level	Post-intervention			McNemar-Bowker (χ²)	*P*-value
	Good knowledge	Moderate knowledge	Poor knowledge		
**Good knowledge**	62 (49.2%)	43 (17.2%)	1 (12.5%)		
**Moderate knowledge**	62 (49.2%)	186 (74.4%)	5 (62.5%)	13.618	.003
**Poor knowledge**	2 (1.6%)	21 (8.4%)	2 (25.0%)		

## Discussion

Recognizing that children’s natural creativity enhances their ability to learn actively, the pan-European e-Bug teaching resource was developed to educate both students and teachers about antimicrobials, hygiene, and how infections are transmitted [15]. In our study, we used the e-Bug module with Grade 12 students and then compared the post-test scores between the different schools, as shown in Table 2, Table 3.

Our study is the first of its kind in our country to assess antimicrobial resistance knowledge among Grade 12 students, as previous local research in this area has primarily focused on healthcare professionals [16]. Our findings align with previous studies showing that the e-Bug module improves AMR knowledge but does not fully correct misconceptions [17]. Similar to the Indian study, we observed significant post-intervention gains yet persistent gaps; however, unlike in India where younger students lacked basic AMR understanding, our older students had strong hygiene knowledge but continued to believe antibiotics treat viral infections. This highlights the need for sustained, context-specific educational strategies alongside curriculum integration [13]. In contrast to the study in Portugal, our assessment was more comprehensive, encompassing questions on immunity, hand hygiene, and microbial characteristics [18].

This study provides the first evidence on the effectiveness of a structured educational intervention to improve AMR knowledge among Grade 12 students in Somalia. The findings demonstrate that the e-Bug module was successful, resulting in a statistically significant improvement in overall knowledge (*P* = 0.003). Our findings on the effectiveness of the e-Bug module are consistent with research from other middle-income settings, such as the study in Manipal, India [13]. Both studies demonstrated that a structured educational intervention significantly improves students’ knowledge regarding antimicrobial resistance. While the Indian study successfully established the module’s effectiveness among younger, junior school students (Class VII), our research extends these findings by confirming its positive impact on older, senior secondary students (Grade 12). This suggests that e-Bug is a robust and adaptable educational tool for a wide range of adolescent age groups.

Our baseline data revealed that while students had a strong grasp of basic hygiene, critical gaps existed in their understanding of microbiology and antibiotic use. The most alarming misconception was the belief that antibiotics are effective against viral infections, a key driver of antibiotic misuse globally [19]. This finding is particularly concerning in the Somali context, where self-medication with antibiotics is rampant [16]. The intervention led to marked improvements in most knowledge areas, most notably a 9.6% increase in students correctly identifying the link between antibiotic misuse and resistance. This demonstrates the module’s capacity to convey complex public health concepts effectively.

However, the intervention‘s impact was not uniform. The deeply ingrained misconception that antibiotics can treat viral illnesses saw only a marginal improvement, with 58.3% of students retaining this incorrect belief post-intervention. This suggests that certain long-held health myths are resistant to change and require more than a single educational session to correct [20,21]. This specific finding underscores the urgent need for continuous, reinforced public health messaging that directly targets this dangerous misunderstanding.

This study has several limitations. The absence of a control group limits the ability to attribute observed changes solely to the intervention. Additionally, knowledge was assessed only in the short term, which restricts conclusions about the sustainability of the observed gains. The use of a convenience sample limits generalizability, and participants were not formally matched by age, sex, or class size, which may introduce bias or confounding, although using each participant as their own control partly mitigates this.

## Conclusion

The e-Bug educational intervention proved to be an effective tool for improving knowledge of antimicrobial resistance among secondary school students in Mogadishu. It successfully addressed several key knowledge gaps and enhanced understanding of the causes of AMR. However, the persistence of the dangerous misconception that antibiotics are effective against viruses highlights the necessity for sustained, multi-pronged educational strategies. We recommend the integration of the e-Bug module into Somalia’s national school curriculum and the development of targeted public health campaigns to foster long-term behavioral change and help curb the growing threat of AMR in the region.

## Declaration of competing interest

The authors report no conflicts of interest in this work.
